# Interplay between Kinase Domain Autophosphorylation and F-Actin Binding Domain in Regulating Imatinib Sensitivity and Nuclear Import of BCR-ABL

**DOI:** 10.1371/journal.pone.0017020

**Published:** 2011-02-11

**Authors:** Martin Preyer, Paolo Vigneri, Jean Y. J. Wang

**Affiliations:** Division of Hematology-Oncology and Moores Cancer Center, Department of Medicine, University of California San Diego School of Medicine, La Jolla, California, United States of America; Wayne State University School of Medicine, United States of America

## Abstract

**Background:**

The constitutively activated BCR-ABL tyrosine kinase of chronic myeloid leukemia (CML) is localized exclusively to the cytoplasm despite the three nuclear localization signals (NLS) in the ABL portion of this fusion protein. The NLS function of BCR-ABL is re-activated by a kinase inhibitor, imatinib, and in a kinase-defective BCR-ABL mutant. The mechanism of this kinase-dependent inhibition of the NLS function is not understood.

**Methodology/Principal Findings:**

By examining the subcellular localization of mutant BCR-ABL proteins under conditions of imatinib and/or leptomycin B treatment to inhibit nuclear export, we have found that mutations of three specific tyrosines (Y232, Y253, Y257, according to ABL-1a numbering) in the kinase domain can inhibit the NLS function of kinase-proficient and kinase-defective BCR-ABL. Interestingly, binding of imatinib to the kinase-defective tyrosine-mutant restored the NLS function, suggesting that the kinase domain conformation induced by imatinib-binding is critical to the re-activation of the NLS function. The C-terminal region of ABL contains an F-actin binding domain (FABD). We examined the subcellular localization of several FABD-mutants and found that this domain is also required for the activated kinase to inhibit the NLS function; however, the binding to F-actin *per se* is not important. Furthermore, we found that some of the C-terminal deletions reduced the kinase sensitivity to imatinib.

**Conclusions/Significance:**

Results from this study suggest that an autophosphorylation-dependent kinase conformation together with the C-terminal region including the FABD imposes a blockade of the BCR-ABL NLS function. Conversely, conformation of the C-terminal region including the FABD can influence the binding affinity of imatinib for the kinase domain. Elucidating the structural interactions among the kinase domain, the NLS region and the FABD may therefore provide insights on the design of next generation BCR-ABL inhibitors for the treatment of CML.

## Introduction

Expression of BCR-ABL is a hallmark of chronic myeloid leukemia (CML), a clonal disease of hematopoietic progenitor cells. The BCR-ABL fusion protein arises from a reciprocal translocation between chromosomes 9 and 22, such that a variable portion of the breakpoint cluster region (*BCR*) gene replaces the first exon of the Abelson murine leukemia virus (*ABL*) proto-oncogene [1], [2]. The kinase activity of the ABL non-receptor kinase is tightly regulated in normal cells [3], [4], [5]. When BCR sequences are fused to ABL, oligomerization through a coiled-coil domain at the N-terminus of BCR [6], [7], [8] plus deletion of the ABL N-terminal CAP region [4], [5] constitutively activate the kinase and unleash its transforming potential [9], [10]. The critical role of the BCR-ABL kinase in CML has been demonstrated by the clinical efficacy of a small molecule inhibitor imatinib mesylate (STI-571, the active ingredient in GleevecTM), that binds to the ABL kinase domain [11], [12], [13], [14]. However, the emergence of imatinib-resistant BCR-ABL in CML patients has called for the development of additional inhibitors and alternative strategies to sustain disease remission [15], [16], [17], [18].

The ABL protein contains three nuclear localization signals (NLS) and a leucine-rich nuclear export sequence (NES) [19], [20], [21]. The normal ABL protein shuttles between the cytoplasm and the nucleus in proliferating cells, and it accumulates in the nucleus when cells are treated with leptomycin B (LMB) [21], [22], [23], [24], an inhibitor of the nuclear export receptor Crm1/exportin-1 [25], [26]. The three NLS and the NES of ABL are present in the BCR-ABL fusion protein. Nevertheless, BCR-ABL is localized exclusively to the cytoplasm [19], [27], [28], [29] and it does not accumulate in the nucleus even after LMB treatment [22]. The inhibition of BCR-ABL kinase with imatinib, however, re-activates nuclear import, leading to nuclear accumulation of this oncoprotein when nuclear export is blocked with LMB [22]. When trapped in the nucleus, BCR-ABL can induce cell death [22], suggesting that the oncogenic activity of BCR-ABL requires its exclusion from the nucleus.

To gain further insights into the inhibition of the NLS function in BCR-ABL, we focused on (a) the inverse correlation between BCR-ABL kinase activity and its nuclear import, and (b) the notion that F-actin-binding is required to retain BCR-ABL in the cytoplasm. We found that the specific mutation of tyrosines 232, 253 and 257 (referring to ABL-1a amino acid numbering), but not six other tyrosine sites in the ABL kinase domain, including Y226 (Y245 in ABL-1b numbering) or Y393 (Y412 in ABL-1b numbering), can abolish the nuclear import of even a kinase-defective BCR-ABL fusion protein. We found that inhibition of the NLS function also involves the C-terminal region of the ABL protein, as a subset of mutations in the F-actin binding domain (FABD) could re-activate the NLS function of kinase-active and autophosphorylated BCR-ABL. However, we also identified other FABD mutations that did not re-activate BCR-ABL nuclear import despite the disruption of their binding to actin filaments. Thus the data presented here suggest that the kinase domain conformation, controlled by three specific tyrosines, and the folding of the C-terminal region, including the FABD, are key determinants in the regulation of the BCR-ABL NLS function.

## Results

### Kinase defective BCR-ABL can be retained in the cytoplasm by kinase-active BCR-ABL

**Figure 1:**
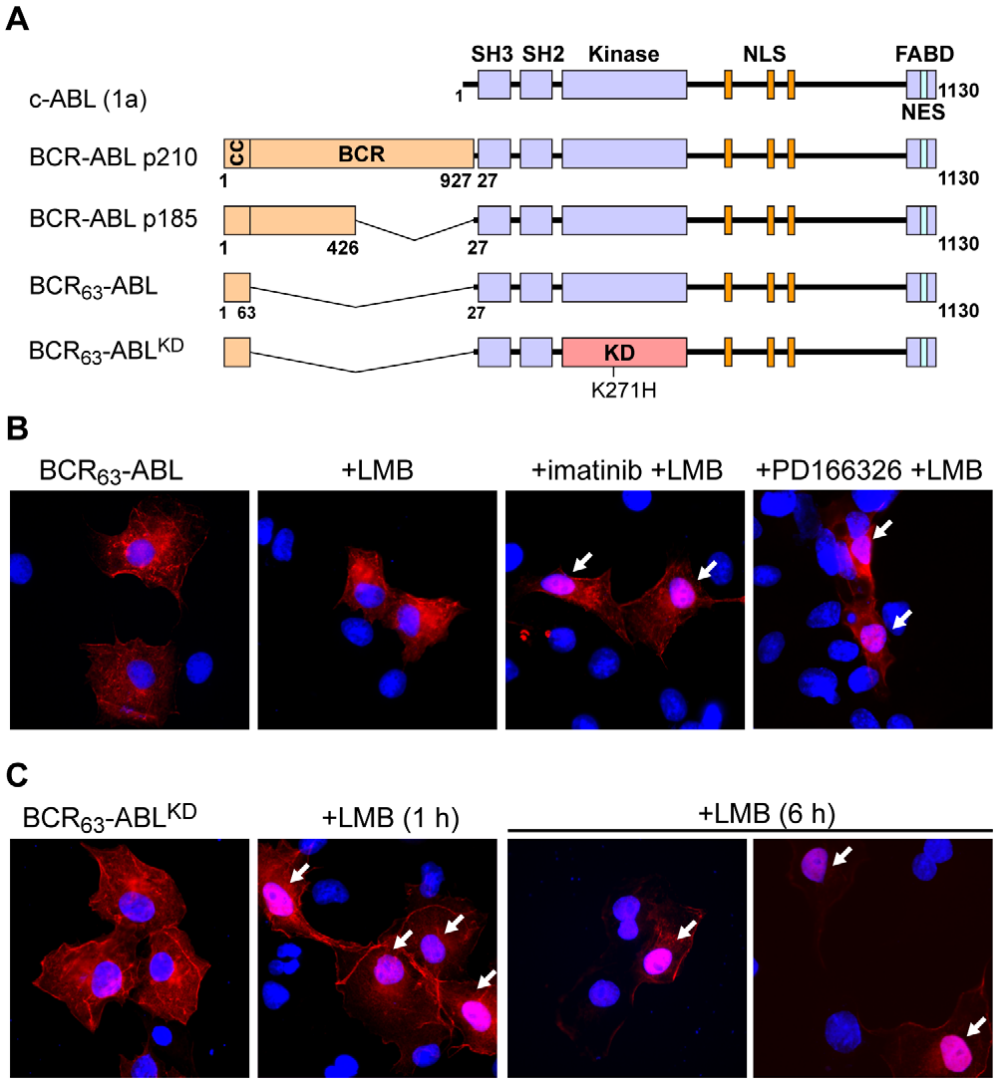
Kinase activity of BCR-ABL inhibits its nuclear import. **A**: Domain structure of ABL, the BCR-ABL p210 and p185 fusion proteins, and the minimal BCR_63_-ABL used in this study. All numbering used herein refers to amino acid positions in the human ABL-1a isoform. The kinase-defective (KD) constructs bear a lysine-to-histidine substitution (K271H) in the ATP-binding site, which renders the kinase catalytically inactive. Abbreviations used are: SH3, src-homology 3; SH2 src-homology 2; FABD, F-actin binding domain; NLS, nuclear localization signal; NES nuclear export signal; cc, coiled-coil oligomerization domain; KD, kinase-defective. **B and C**: COS cells ectopically expressing active BCR_63_-ABL (**B**) or the kinase-defective mutant (BCR_63_-ABL^KD^) (**C**) were treated with the CRM1-inhibitor LMB (10 nM) for either 1 or 6 hours, which leads to accumulation in the nuclei of cells only if the protein is imported. The presence of nuclear staining in LMB-treated cells demonstrates that the protein is imported. Cells displaying notable nuclear staining (resulting from nuclear import) of BCR-ABL are marked with white arrows. The BCR-ABL kinase activity was also blocked by treatment with the kinase inhibitors imatinib (10 µM) or PD166326 (10 nM) for 16 hours to enable nuclear import. BCR-ABL localization was determined by immunofluorescence staining with an anti-ABL antibody (8E9, shown in red). The endogenous ABL was not observed under the experimental conditions, which were designed to detect only the ectopically expressed proteins that were present at a much higher abundance than the endogenous ABL protein. DNA is counterstained in blue with Hoechst dye.

We have previously shown that the BCR-ABL protein can accumulate in the nucleus after the combined treatment with imatinib that inhibits its kinase activity and LMB that inhibits Crm1/exportin-1 to block nuclear export [22]. With a kinase-defective (KD) BCR-ABL mutant, generated by substitution of the critical Lysine in the ATP-binding site, nuclear accumulation is achieved by treatment with LMB alone, suggesting that the NLS is active in BCR-ABL^KD^ [22] (supplementary Figure S1). We have examined the contribution of BCR sequences to the inhibition of BCR-ABL nuclear import, specifically focused on the BCR Tyr^177^ phosphorylation site and the BCR R^91^ASASRP^97^ region that binds to the adaptor protein 14-3-3-delta, because these BCR sequences mediate protein-protein interactions that might contribute to the cytoplasmic retention of BCR-ABL. We have found that BCR-ABL-Y177F and BCR-ABL-Δ91-97 are localized exclusively to the cytoplasm even after LMB treatment (supplementary Figure S1), suggesting that pY177 and 14-3-3-binding are not the major determinants for the cytoplasmic localization of BCR-ABL. Previously, we have shown that fusion of the N-terminal 63 amino acids, which contain a coiled-coil oligomerization domain, is sufficient to activate the BCR-ABL kinase and cause its nuclear exclusion [6], [28]. To investigate how the activated kinase causes the inhibition of its NLS function, we conducted this study with a BCR_63_-ABL fusion protein containing only the N-terminal BCR oligomerization domain that is necessary and sufficient for the constitutive activation of the BCR-ABL kinase activity [6], [7] (Figure 1A).

The BCR_63_-ABL fusion protein is present in the cytoplasm of COS cells (Figure 1B) and *Abl-null* 3T3 fibroblasts (not shown), but accumulates in the nucleus following the combined treatment with imatinib and LMB (Figure 1B). The subcellular localization of BCR_63_-ABL and its response to imatinib and LMB are therefore similar to that of p210- and p185-BCR-ABL [22]. The nuclear accumulation of BCR_63_-ABL was also achieved with the combined treatment of LMB plus PD166326, which is another ABL kinase inhibitor (Figure 1B). Binding of PD166326 and imatinib to the ABL kinase domain requires the “DFG-Asp out” conformation of the kinase N-lobe [30]. However, the catalytic site conformation, particularly the activation loop and the helix αC of PD166326- and imatinib-bound ABL kinase domains are not identical [4], [31]. It thus appears that the configuration of the activation loop and helix αC may not be important to the regulation of the NLS function. On the other hand, as to be shown below, the “DFG-Asp out” conformation imposed by binding to imatinib or PD166326, is likely to be critical to the regulation of the NLS function.

**Figure 2:**
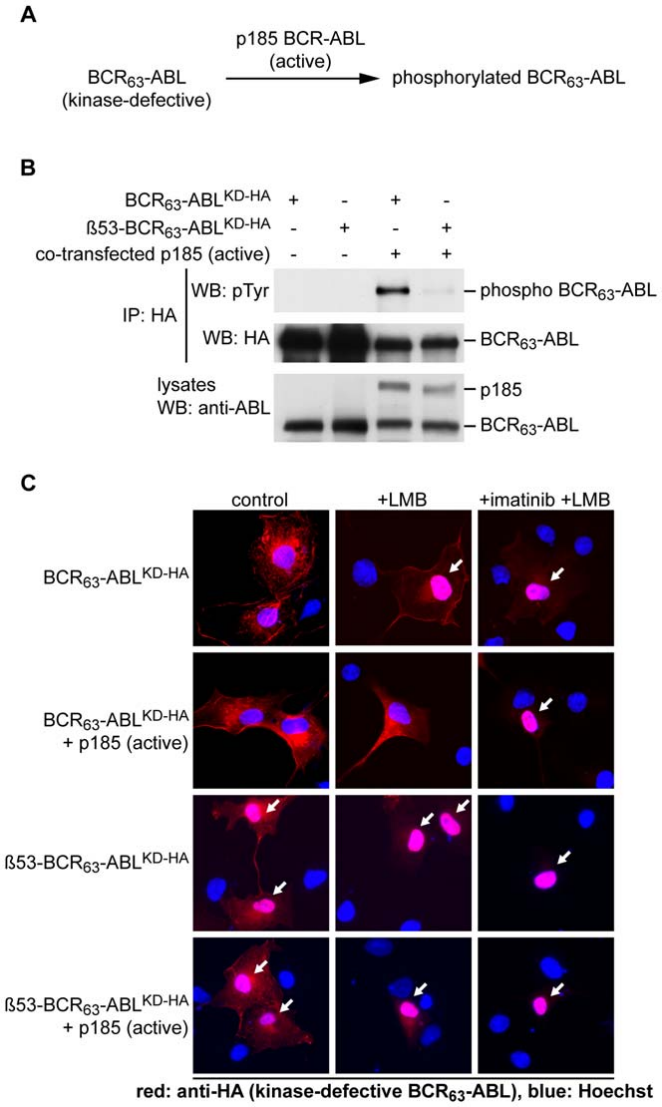
Trans-phosphorylation of kinase-defective BCR-ABL blocks its nuclear import. **A**: Scheme of experimental design. Kinase-defective BCR_63_-ABL constructs were co-transfected with kinase active p185-BCR-ABL to induce tyrosine phosphorylation of the kinase-defective protein. **B**: BCR_63_-ABL^KD^ constructs were immunoprecipitated with an anti-HA antibody from COS cells that were co-transfected with the indicated plasmids. Immunoblots from HA-pulldowns (top) and total cell lysates (bottom) were probed with the indicated antibodies to detect the tyrosine phosphorylation of BCR_63_-ABL^KD^. The previously described β53-BCR_63_-ABL^KD^ has a beta-turn inserted at position 53, which disables the coiled-coil oligomerization domain [6]. **C**: COS cells were transfected with the indicated HA-tagged, kinase-defective BCR_63_-ABL^KD^ constructs either alone or in co-transfection with a kinase-active p185-BCR-ABL. The localization of the kinase-defective BCR_63_-ABL proteins was detected by immunostaining with an anti-HA antibody (red).

The kinase-defective BCR_63_-ABL^KD^, which is catalytically inactive through Lys271His (Lys290 in ABL-1b numbering) substitution in the kinase domain [32], was predominantly cytoplasmic in COS cells (Figure 1C), but became partially nuclear after 1 hour LMB treatment (Figure 1C) and mostly nuclear after 6 hours LMB exposure (Figure 1C and 2C). This demonstrates that BCR_63_-ABL^KD^, similar to BCR-ABL^KD^ [22](supplementary Figure S1), can undergo nucleo-cytoplasmic shutting, and the continuous nuclear import allows its nuclear accumulation when export is blocked by LMB.

To determine if autophosphorylation is responsible for inhibiting the NLS function, we co-expressed p185-BCR-ABL with BCR_63_-ABL^KD^ to allow trans-phosphorylation of the kinase-defective protein via oligomerization through the BCR coiled-coil (Figure 2B). When co-expressed with p185-BCR-ABL, the BCR_63_-ABL^KD^ protein became tyrosine phosphorylated and did remain cytoplasmic after LMB treatment, as revealed by immunofluorescence against the HA-tag present only in the BCR_63_-ABL^KD^ protein (Figure 2C). Inhibition of the co-expressed p185-BCR-ABL kinase with imatinib re-activated the nuclear import of BCR_63_-ABL^KD^, indicated by its nuclear accumulation in response to LMB. We then repeated these experiments with β53-BCR_63_-ABL^KD^, which has a β-turn inserted at position 53 to disrupt the coiled-coil oligomerization domain [6]. Co-expression with p185-BCR-ABL induced a very low level of phosphotyrosine in the β53-BCR_63_-ABL^KD^ (Figure 2B), and correspondingly, it did not inhibit the nuclear import of β53-BCR_63_-ABL^KD^ (Figure 2C). We also found that p185-BCR-ABL did not affect the subcellular localization of ABL, which does not become tyrosine phosphorylated and showed continuous nuclear-cytoplasmic shuttling (supplementary Figure S2). These results suggest that tyrosine phosphorylation of BCR_63_-ABL, rather than its catalytic activity *per se*, can lead to the inhibition of its nuclear import.

### Mutation of Y232, Y253 and Y257 in the ABL kinase N-lobe blocks nuclear import

**Figure 3:**
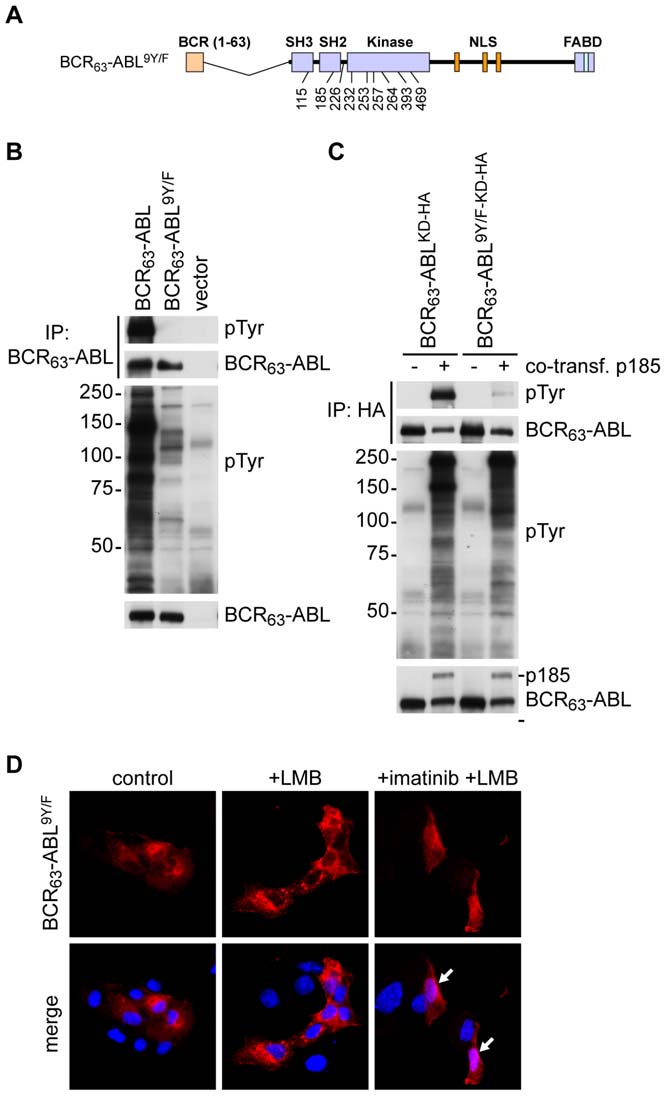
Mutation of nine tyrosines in BCR_63_-ABL does not restore nuclear import. **A**: In the BCR_63_-ABL^9Y/F^ protein, nine autophosphorylation sites are mutated to phenylalanines. The position and amino acid number (according to that of ABL-1a) of the Tyr/Phe (Y/F) substitutions are indicated in the schematic drawing. **B**: The BCR_63_-ABL^9Y/F^ protein and the BCR_63_-ABL protein were immunoprecipitated from transfected cells. The levels of phosphotyrosine and the BCR_63_-ABL protein were detected by immunoblotting from immunoprecipitates (top) and whole cell lysates (bottom) using monoclonal antibodies 4G10 (for phosphotyrosine) and 8E9 (for ABL). **C**: HA-tagged kinase-defective BCR_63_-ABL or a corresponding 9Y/F-mutant were co-transfected with kinase-active p185-BCR-ABL to allow for trans-phosphorylation. The kinase-defective proteins were immunoprecipitated using an anti-HA antibody, and immunoblotted as in (B). **D**: The phosphorylation site mutant BCR_63_-ABL^9Y/F^ was transfected in COS cells and its localization determined by immunofluorescence after treatment with 10 nM LMB for 6 hours, or 10 µM imatinib and LMB. Nuclear localization was only observed after treatment with imatinib and LMB, as indicated by the solid white arrows.

**Figure 4:**
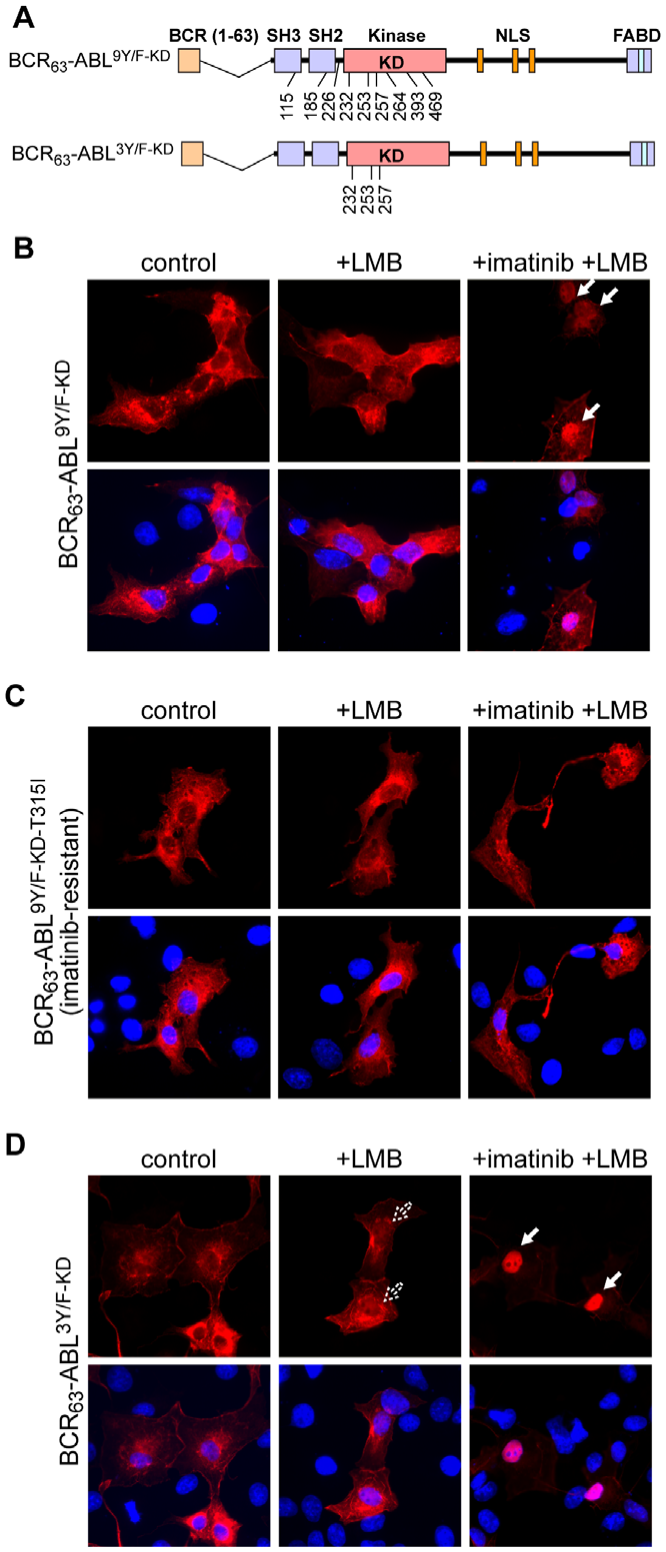
Mutation of Tyr232, Tyr253, and Tyr257 to phenylalanine blocks nuclear import of kinase-defective BCR_63_-ABL. **A**: Schematic representation of BCR_63_-ABL^9Y/F-KD^ and BCR_63_-ABL^3Y/F-KD^ constructs. The constructs encode catalytically inactive proteins due to the KD mutation (Lys271His) in the kinase domain. The position and amino acid number of the 9Y/F and the 3Y/F substitutions are indicated in the scheme according to the ABL-1a amino acid numbering. **B**: The kinase-defective BCR_63_-ABL^9Y/F-KD^ construct was transfected into COS cells and the localization of the protein determined by immunofluorescence. Nuclear localization of the kinase-defective protein was only observed in cells treated with 10 µM imatinib and 10 nM LMB, as indicated by the white arrows. **C**: The gatekeeper mutation (T315I), which prevents imatinib from binding to the ATP binding pocket of BCR-ABL, was introduced into the BCR_63_-ABL^9Y/F-KD^ backbone to test whether imatinib enables the nuclear import of the kinase-defective protein through direct binding to its kinase domain. The respective construct (BCR_63_-ABL^9Y/F-KD-T315I^) was transfected into COS cells and the subcellular localization of the ectopically expressed protein examined by immunofluorescence. No nuclear localization of the protein was detected in the absence or presence of imatinib and LMB. **D**: The BCR_63_-ABL^3Y/F-KD^ protein, in which the three tyrosines Y232, Y253, and Y257 are mutated to phenylalanines, was expressed in COS cells. The subcellular localization was again determined by immunofluorescence in untreated cells, as well as after the treatment with imatinib and LMB. Dashed arrows indicate minimal nuclear staining in the absence of imatinib, white arrows point to cells showing predominately nuclear localization in the presence of imatinib.

A total of nine tyrosines within the ABL-portion of BCR-ABL have been shown to be phosphorylated by tandem mass spectrometry analysis [33], [34]. To further address the role of autophosphorylation in the regulation of the NLS function, we mutated those nine tyrosines to phenylalanines creating a mutant termed BCR_63_-ABL^9Y/F^ (Figure 3A). Indeed, this 9Y/F-mutant was poorly autophosphorylated (Figure 3B), and was weakly phosphorylated in *trans* by p185-BCR-ABL (Figure 3C). Thus, if the hypothesis that autophosphorylation blocks nuclear import were correct, the 9Y/F-mutant protein would be expected to undergo nuclear import. Surprisingly, we found that the BCR_63_-ABL^9Y/F^ protein did not accumulate in the nucleus after LMB treatment. Even more surprising was the observation that imatinib treatment still induced the nuclear import of this BCR_63_-ABL^9Y/F^ fusion protein (Figure 3D). We then created a kinase-defective version of the 9Y/F-mutant and found that the BCR_63_-ABL^9Y/F-KD^ fusion protein also failed to undergo nuclear import (Figure 4B). Furthermore, imatinib could again override the 9Y/F effect and induce nuclear import of BCR_63_-ABL^9Y/F-KD^ (Figure 4B). To determine whether the stimulatory effect of imatinib on the nuclear import of the 9Y/F-KD-mutant was indeed caused by binding of the drug to the mutant protein, we introduced another amino acid substitution, T315I, which confers imatinib-resistance through interference with drug binding [18]. Imatinib did not stimulate the nuclear import of BCR_63_-ABL^9Y/F-KD-T315I^ (Figure 4C), showing that the direct binding of this drug to the kinase domain is required to reactivate the NLS function of the 9Y/F mutant.

**Figure 5:**
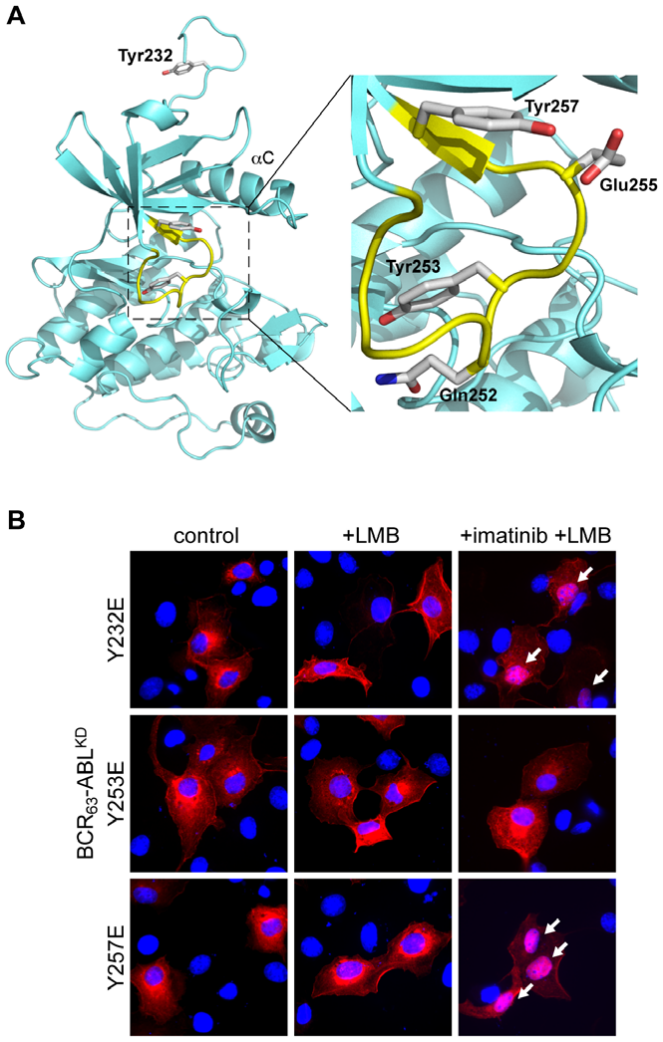
Phosphomimetic mutation of either Tyr232, Tyr253, or Tyr257 blocks nuclear import of kinase-defective BCR_63_-ABL. **A**: Position of tyrosines 232, 253, and 257 as seen in a crystal structure of the ABL kinase domain bound to imatinib ([31], PDB code 1IEP). The Tyr253 and Tyr257 are in the P-loop (yellow), and are engaged in interactions with other P-loop side chains (Gln252 and Glu255) in this structure. Tyr232 is located in the SH2-kinase linker region and situated right above the kinase N-lobe in this structure. **B**: BCR_63_-ABL^KD^, in which either Tyr232, 253 or 257 was mutated to glutamic acid, was transiently expressed in COS cells. The localization of each of these three Y/E-mutant proteins after the indicated treatments with LMB and imatinib was determined by immunofluorescence. Merged images of BCR-ABL staining (red) and DNA (blue) are shown. Cells displaying nuclear staining are marked by the white arrows.

To identify which of the nine Y/F mutations was responsible for the inhibition of nuclear import, we systematically reverted the nine phenylalanines back to tyrosines. We found that reversion of three phenylalanines at positions 232 (SPN**Y^232^**D), and 253, 257 (GGGQ**Y^253^**GEV**Y^257^**EG) restored nuclear import, i.e., the BCR_63_-ABL^6Y/F-KD^ protein could accumulate in the nucleus by the treatment with LMB alone without imatinib (supplementary Figure S3). Conversely, mutation of tyrosines 232, 253 and 257 to phenylalanines was sufficient to block nuclear import, i.e., the BCR_63_-ABL^3Y/F-KD^ required the combined treatment with imatinib and LMB to accumulate in the nucleus (Figure 4D). Single and double mutants, having either one or two of the three tyrosines mutated to phenylalanines, also showed some nuclear import, indicated by weak nuclear accumulation in LMB-treated cells (supplementary Figure S4). We then mutated the three critical tyrosines (Tyr232, 253 or 257) individually to glutamic acid, which mimics phosphorylation, in the BCR_63_-ABL^KD^ context and found that each Y/E substitution alone is sufficient to block the nuclear import of this kinase-defective BCR_63_-ABL^KD^ protein (Figure 5B). Again, treatment with imatinib induced nuclear accumulation of the phosphomimetic mutants Y232E and Y257E (Figure 5B). However, imatinib did not stimulate the nuclear import of the Y253E mutant (Figure 5B), which is consistent with the fact that BCR-ABL^Y253H^ was an imatinib-resistant mutant isolated from drug-resistant CML cells [35].

Results shown in Figures 3, 4 and 5 suggest that Tyr232, Tyr253 and Tyr257 play crucial roles in regulating the NLS function. Because the phosphomimetic mutation of any of these three tyrosines to glutamic acid is sufficient to inhibit the nuclear import of a kinase-defective BCR_63_-ABL^KD^, phosphorylation of any of these three tyrosines is likely to block the NLS function. The unexpected finding that mutations of these three tyrosines to phenylalanines also affected the NLS function lends additional support to the notion that these three tyrosines in the ABL kinase domain are involved in the regulation of nuclear import. Tyr253 and Tyr257 are in the P-loop of the kinase N-lobe, and their hydroxyl side-chain interactions with neighboring amino acids contribute to the P-loop conformation in the current crystal structure (Figure 5A). Tyr232 is in the SH2-kinase linker, and the X-ray structure of the ABL kinase domain shows it to be located next to the kinase N-lobe with its hydroxyl side-chain being solvent-exposed (Figure 5A). It is interesting to find that imatinib-binding, which locks the kinase N-lobe in the “DFG-Asp out” conformation [30], can override the negative effect of these Y/F mutations on the NLS function. Together, these results suggest that autophosphorylation occurring at specific tyrosines in the SH2-kinase linker (Y232) and the kinase P-loop (Y253, 257) can affect the N-lobe conformation, which controls the NLS function.

### Regulation of BCR-ABL nuclear import by the F-actin binding domain

**Figure 6:**
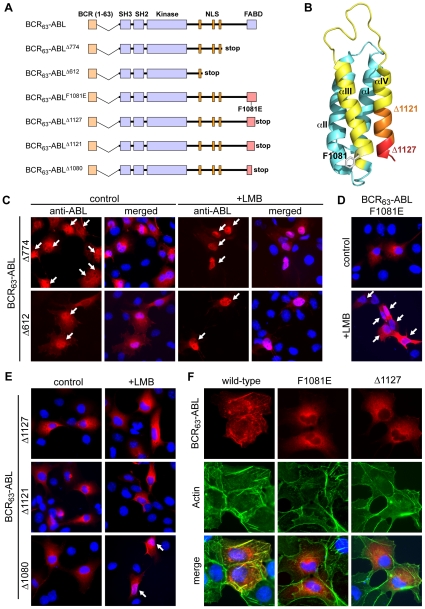
The FABD but not binding to actin filaments is required to block import of BCR_63_-ABL. **A**: Schematic representation of the constructs used. BCR_63_-ABL was either truncated by introducing a stop codon at the indicated amino acid positions or a point mutation (F1081E) within helix-3 of the actin-binding domain (FABD) to abolish binding to filamentous actin. **B**: NMR structure of the FABD of ABL ([38], PDB code 1ZZP). The C-terminal residues that are deleted in the truncation mutants Δ1080, Δ1121 and Δ1127 are highlighted in yellow, orange, and red, respectively. The phenyl side-chain of F1081 in helix-3 (αIII), which was mutated to glutamic acid, is shown in white. **C, D and E**: COS cells expressing the indicated BCR_63_-ABL mutants were left untreated or treated with LMB as indicated. Merged images of anti-ABL staining show the respective mutant BCR_63_-ABL in red and DNA in blue. Cells showing nuclear BCR_63_-ABL staining are marked by the white arrows. **F**: COS cells were transfected with BCR_63_-ABL or the indicated mutants and processed for immunofluorescence. Images of anti-ABL staining (red) and F-actin counterstained with Alexa-488-conjugated phalloidin (green) are shown individually, and merged with DNA (blue) images. Co-localization of BCR_63_-ABL with actin fibers results in yellow color in the merged images.

The oligomerization of BCR-ABL also stimulates its association with F-actin stress fibers and cortical actins through an F-actin binding domain (FABD) at the C-terminus of ABL [28], [36], [37]. The NMR structure of the FABD (aa-998 to aa-1130) shows a four-helix bundle (Figure 6B), which is also found in several other F-actin binding proteins such as vinculin and talin [38], [39]. It has previously been proposed that tethering to F-actin is the predominant mechanism for the cytoplasmic retention of the ABL protein [38]. Because the BCR_63_-ABL fusion protein is localized to actin filaments [36], we made a series of C-terminal deletions in the BCR_63_-ABL backbone to disrupt the FABD helix-4 (Δ1127, Δ1121), the FABD helices-3 & 4 (Δ1080), the entire FABD (Δ774), or the FABD plus the second and third NLS (Δ612) (Figure 6A). We also mutated F1081 in the FABD helix-3 to glutamic acid in BCR_63_-ABL because this single substitution mutation can also inhibit F-actin binding [38]. The subcellular distribution of these F-actin binding defective mutants was then examined in the absence or presence of LMB. In the absence of drug treatment, the BCR_63_-ABLΔ^612^ (containing only NLS1) and the BCR_63_-ABLΔ^774^ (containing NLS1, 2 and 3) proteins were already localized diffusely throughout the cytoplasm and the nucleus (Figure 6C), a subcellular distribution similar to that of the full-length ABL protein, which undergoes continuous nuclear import and export [21] (supplementary Figure S2). These results suggest that nuclear import of BCR_63_-ABL can be restored when the C-terminal region beyond NLS-3 is deleted.

**Figure 7:**
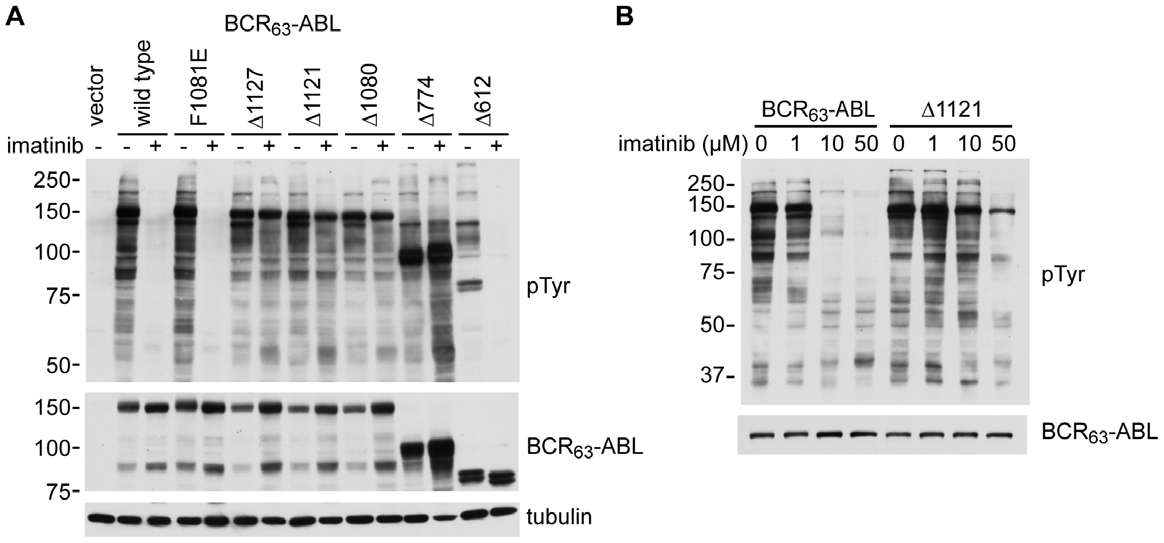
Kinase activity and imatinib-sensitivity of BCR_63_-ABL mutants. **A and B**: COS cells were transfected with BCR_63_-ABL or the indicated mutant constructs. The cells were left untreated or treated with 10 µM imatinib (**A**) or different doses of imatinib (**B**) for 16 hours to inhibit BCR-ABL kinase activity. Immunoblotting of whole cell lysates with an antibody (4G10) against phophostyrosine (pTyr) was used to indicate the levels of the BCR-ABL tyrosine kinase activity. The levels of the BCR_63_-ABL protein were determined by immunoblotting with an anti-ABL antibody (8E9). The levels of tubulin were shown as a loading control. The positions of the molecular weight markers (in kilodalton) are indicated at the left of the blot.

Unlike the Δ774 deletion, mutations within the FABD (aa-998 to aa-1130) exerted variable effects on the NLS function. The BCR_63_-ABL^F1081E^ mutant, which does not associate with F-actin (Figure 6F), remains cytoplasmic in the absence of drug treatment (Figure 6D). However, nuclear localization of a fraction of the BCR_63_-ABL^F1081E^ protein was observed following treatment with LMB alone without the need of kinase inhibition by imatinib (Figure 6D), suggesting that the NLS function in BCR_63_-ABL^F1081E^ is partially restored despite autophosphorylation (Figure 7A). The BCR_63_-ABLΔ^1080^ mutant behaved similarly to the F1081E mutant in that its nuclear localization can be stimulated by treatment with LMB alone (Figure 6E). The behavior of these two mutant proteins, with defects of helix-3 in the FABD, suggested that binding to F-actin might be responsible for the inhibition of nuclear import. At odds with this interpretation, however, was the behavior of two other FABD deletion mutants, Δ1127 and Δ1121. These two mutant proteins do not bind F-actin either (Figure 6F, supplementary Figure S5) due to defects in the highly conserved helix-4, which is essential to the binding of F-actin [28], [36], [38], [40]. However, Δ1127 and Δ1121 did not accumulate in the nucleus when treated with LMB alone (Figure 6E), showing that the NLS function in these two mutants remained inhibited despite the loss of F-actin binding.

These results suggest that the C-terminal region beyond NLS-3, and including helix-3 of the FABD, also plays an important role in the inhibition of the NLS function. When this C-terminal region is deleted, or when the FABD helix-3 is mutated, the NLS function can be restored despite an active kinase and autophosphorylation. However, the direct binding to F-actin *per se* is not required for the inhibition of nuclear import, as indicated by the inability of the FABD helix-4 mutants (Δ1121, Δ1127) to re-activate the NLS function.

### C-terminal truncation affects the sensitivity of BCR-ABL to imatinib

To rule out the possibility that C-terminal mutations might affect the levels of autophosphorylation, we measured the reactivity of total lysates with the monoclonal anti-phosphotyrosine (pTyr) antibody (4G10) from cells ectopically expressing the different C-terminal mutants. The major pTyr band in each of the whole cell lysates was BCR_63_-ABL itself (Figure 7A). When normalized to the protein levels, the steady state levels of tyrosine phosphorylation were not significantly altered by any of the C-terminal mutations (Figure 7A). Thus, the imatinib-independent nuclear import of the F1081E and the Δ1080, Δ774, Δ612 mutants occurred despite their kinase activity and autophosphorylation. Since *either* the binding of imatinib to the kinase N-lobe *or* the deletion of the C-terminal region beyond NLS-3 was sufficient to re-activate the NLS function, these results suggest that the kinase domain autophosphorylation and the C-terminal region including the FABD are both required to inhibit the NLS function in the kinase-active BCR_63_-ABL protein.

Given the finding that the kinase domain conformation and the FABD are both involved in the regulation of the NLS function, we tested whether C-terminal mutations might affect the kinase sensitivity to imatinib, which only binds to one of three conformations, i.e., the “DFG-Asp out”, of the kinase N-lobe [30]. We treated cells with a saturating concentration of imatinib (10 µM, 16 h) and found comparable inhibition of tyrosine phosphorylation of BCR_63_-ABL, the F1081E, and the Δ612 mutants (Figure 7A). By contrast, four other deletion mutants (Δ1127, Δ1121, Δ1080, Δ774) were less sensitive to inhibition by imatinib (Figure 7A). The imatinib dose-response was further examined with the Δ1121 mutant, which showed approximately a 10-fold reduced sensitivity to imatinib when expressed at the same level as BCR_63_-ABL (Figure 7B). The smallest deletion that caused increased resistance to imatinib is Δ1127, which lacks only the last four amino acids in the helix-4 of the FABD (Figure 6B). Helix-4 of the FABD is also missing in the other three deletions, Δ1121, Δ1080, Δ774, that exhibited resistance to imatinib (Figure 7A). However, the deletion mutant Δ612, which lacks the FABD, the NLS-2 and the NLS-3, was sensitive to imatinib at a level comparable to the un-mutated BCR_63_-ABL (Figure 7A). Because the three-dimensional structural information of the full-length ABL is not available at this time, we could only interpret these results to suggest that the three different kinase N-lobe conformations [30] may be subjected to modulation by the ABL C-terminal region involving the NLS-2, the NLS-3 and the helix-4 of the FABD.

## Discussion

### The kinase domain conformation regulates BCR-ABL nuclear import

**Figure 8:**
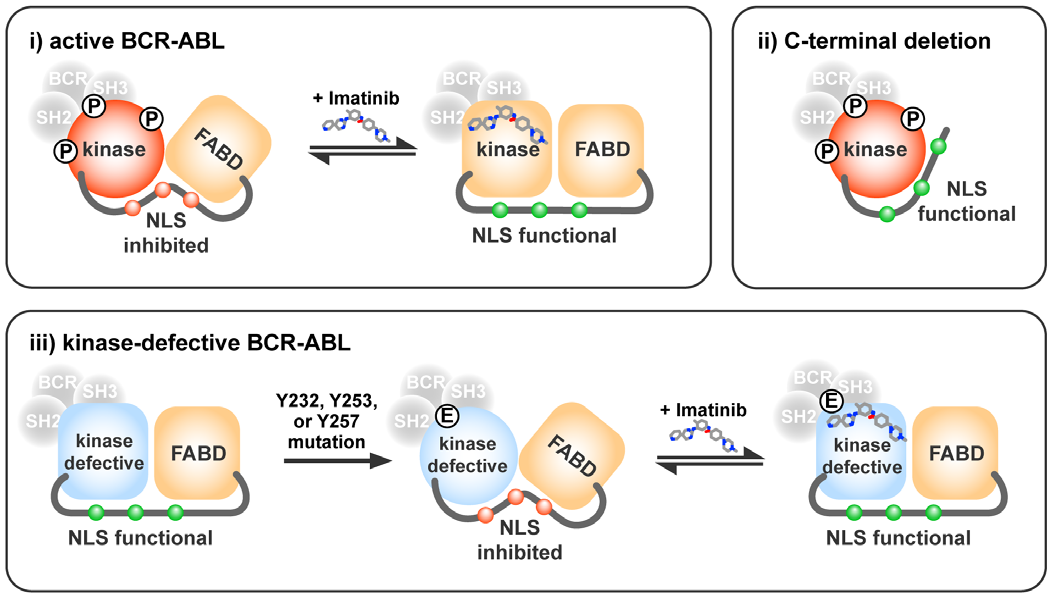
A model for the regulation of BCR-ABL nuclear import through conformational interplay between the kinase domain, the FABD and the NLS region. (i) Tyrosine phosphorylation at Y232, Y253 or Y257 causes the kinase domain to adopt a conformation that affects the folding of the C-terminal region and leading to the inhibition of the NLS function (indicated by the red color of the three nuclear localization signals depicted as small circles embedded in a proline-rich linker between the kinase domain and the F-actin binding domain, FABD). The kinase domain autophosphorylation-induced occlusion of the NLS also requires the C-terminal region beyond the third NLS (NLS-3) and including an intact helix-3 of the FABD. Binding of imatinib reverts the kinase domain back to the “DFG-Asp out” N-lobe conformation that alters the folding of the C-terminal region to un-mask the NLS (indicated by the green color of the three nuclear localization signals). (ii) Deletion of C-terminal sequences beyond the NLS-3 unmasks the NLS despite the kinase domain autophosphorylation. (iii) Mutation of Y232, Y253 or Y257 to glutamic acid (E) also alters the kinase domain conformation to trigger the inhibition of the NLS function. The NLS-inhibitory effect of the tyrosine to glutamic acid substitutions can be observed in a kinase-defective BCR_63_-ABL. Binding of imatinib induces the “DFG-Asp out” conformation of the kinase domain [30], and this imatinib-bound conformation can override the effect of the glutamic acid substitution to re-activate the NLS function.

It is well established that the activated BCR-ABL kinase activity is responsible for the inhibition of its nuclear import [19], [22], [28]. Because BCR-ABL kinase phosphorylates itself and many cellular proteins [33], it is possible to imagine a variety of mechanisms for the inhibition of its NLS function. Results from this study suggest that the activated BCR-ABL kinase oligomer inhibits its NLS function through autophosphorylation and requires an intact C-terminal region including the FABD, but not the binding to F-actin, to block nuclear import (Figure 8).

By narrowing the investigation of autophosphorylation sites to the BCR_63_-ABL protein, we have identified three tyrosines in the kinase domain to play a role in the regulation of the NLS function. Previous studies have shown that phosphorylation of Tyr226 (Tyr245 in ABL-1b numbering) in the SH2-kinase linker and Tyr393 (Tyr412 in ABL-1b numbering) in the activation loop can stimulate ABL kinase activity by disrupting the auto-inhibitory assembly of the SH3/SH2/kinase domains [41], [42], [43]. We have found that mutations of Tyr226, Tyr393 and four other phosphorylation sites (Tyr115, Tyr185, Tyr264, Tyr469, in ABL-1a numbering) did not have any detectable effect on the NLS function. Instead, we found that mutations of three tyrosines (Tyr232, Tyr253 and Tyr257) in the SH2-kinase linker and the kinase P-loop cause a dominant inhibition of the NLS function even in a kinase-defective BCR_63_-ABL. Phosphomimetic mutation of any one of those three tyrosines to glutamic acid is sufficient to inhibit the NLS function. In addition, triple mutations of all three tyrosines to phenylalanines (3Y/F) also inhibit the NLS function even in a kinase-defective BCR_63_-ABL. Interestingly, direct binding to imatinib can restore the NLS function in the 3Y/F mutant. These results suggest that the conformation of the kinase domain, particularly its N-lobe, plays a critical role in the regulation of the NLS function. Drawing on the X-ray structures showing that the kinase domain can adopt one of three stable configurations [30], it appears that the NLS is only active when the kinase is in the “DFG-Asp out” configuration, which is stabilized by imatinib binding. Phosphorylation at any of these three tyrosines may shift the N-lobe away from the “DFG-Asp out” conformation and thus resulting in the inhibition of the NLS function. Removal of the hydroxyl side-chains of these three tyrosines may also alter the N-lobe conformation to inhibit the NLS function.

### Regulation of BCR-ABL nuclear import by the FABD

Because BCR-ABL binds F-actin and co-localizes with actin filaments in cells [28], [36], [37], it has been suggested that BCR-ABL is tethered to F-actin and hence not imported into the nucleus [38]. Indeed, we show here that C-terminal mutations, including those that disrupt helix-3 of the FABD, can restore the NLS function even under conditions when the BCR_63_-ABL kinase is active and autophosphorylated. However, we found that deletion of helix-4 of the FABD (Δ1127 and Δ1121) was unable to release the block on the NLS function. Because helix-4 mutations disrupt the F-actin binding function of the FABD, these results show that F-actin binding *per se* is not required for the inhibition of the NLS function. Rather, the C-terminal region beyond the NLS-3, including the integrity of helix-3 of the FABD, is required for the activated BCR_63_-ABL kinase conformation to induce a blockade of the NLS function.

Taken together, our results can be accommodated by a model where the kinase domain conformation may affect the folding of the C-terminal region including the FABD to regulate the NLS function. As illustrated in Figure 8, which represents but one of several possible scenarios, the activated kinase conformation with autophosphorylation at one of three specific tyrosine sites can influence the folding of the C-terminal region to mask the three NLS through interactions that involve an intact FABD helix-3. The imatinib-bound kinase conformation causes a change in the folding of the C-terminal region, leading to the un-masking of the three NLS.

The interplay between the kinase N-lobe conformation and the FABD is also supported by the results that FABD mutations can affect the kinase sensitivity to imatinib. In the absence of three-dimensional structural data, we can only imagine how the FABD and the region between aa-612 and aa-774, which contains the NLS-2 and NLS-3, might influence the kinase N-lobe conformation. It appears that disruption of the FABD helix-4 can shift the equilibrium of the kinase N-lobe towards those conformations that do not bind imatinib and thus causing imatinib resistance through a mechanism that also requires the sequences surrounding the NLS2 and the NLS-3 region (aa-612 to aa-774). The precise understanding of the conformational interactions among the kinase domain, the NLS region, and the FABD will await the elucidation of the three-dimensional structure of the BCR-ABL or the full-length ABL protein.

### Experimental Procedures

#### Cell culture and reagents

The simian kidney cell line COS1 (American Type Culture Collection) were cultured in DMEM medium supplemented with 10% fetal bovine serum. Transfection of cells was performed with FuGENE6 (Roche Biochemical Inc.) according to the manufacturer's instruction. BCR-ABL kinase inhibitors imatinib (10 µM) or PD166326 (10 nM) were used for 16–24 hours to inactivate BCR-ABL kinase. Leptomycin B (LMB, Kosan Bioscience Inc.) was added at a final concentration of 10 nM for the last 6 hours before fixation or as indicated.

#### Plasmid construction

The BCR_63_-ABL, the BCR_63_-ABLΔ^612^ [28], and the β53-BCR_63_-ABL [6] have been described. C-terminal truncations were made by PCR-based methods as previously described [40]. Point mutations were created by two-step PCR-based mutagenesis, and constructs were sequenced for amplification errors. GFP fusion proteins were made with peGFP-c1 (Stratagene) by PCR-based methods.

#### Immunofluorescence

Cells were seeded onto cover slips and transfected with the specified expression plasmids 24 hours later. Cells were fixed 24 hours after transfection in 4% formaldehyde, permeabilized with 0.3% Triton X-100 in phosphate-buffered saline (PBS), blocked with PBS/10% normal goat serum, and incubated with monoclonal antibodies HA.11 against the hemagglutinin tag (Covance), or anti-ABL (8E9) at a concentration of 1 µg/ml in blocking solution for 1 hour at room temperature. Cells were then incubated with Alexa Fluor 568-conjugated goat secondary antibodies (Molecular Probes) and Alexa Fluor 488-conjugated phalloidin (Molecular Probes) for 1 hour. Nuclei were counterstained with Hoechst 33258 (Molecular Probes) and coverslips mounted onto glass slides with gel mount (Biomeda). Epifluorescence microscopy was performed with a Nikon microscope and images were digitally acquired with a 0.60X HRD060-NIK CCD camera (Diagnostic Instruments). Experiments were performed at least twice for each construct. The localization of each of the BCR-ABL constructs was evaluated in 50–100 transfected cells across the slide. The NLS function in a construct was scored as being inactive when no nuclear localization was observed after the combined treatment with imatinib and LMB. When nuclear signal was observed with a construct, either before or after the single or the combined drug treatments, the nuclear localization was typically seen in the majority of cells, and representative images were taken and shown here.

#### Immunoprecipitation and Immunoblotting

Cell lysates were prepared in radio-immunoprecipitation assay buffer (150 mM NaCl, 50 mM Tris pH 7.2, 0.1% SDS, 1.0% NP-40, 0.25% sodium deoxycholate, 1 mM EDTA, 1 mM Na_3_VO_4_, 1 mM NaF, 10 mM sodium β-glycerophosphate). For immunoprecipitations 250 µg of total protein were incubated with 1 µg antibody (HA.11) for two hours and immune complexes were captured with 30 µl protein-G Sepharose beads (Amersham Pharmacia Biotech) for 1 hour at 4°C. Immunoprecipitates were fractionated by SDS-PAGE and transferred to polyvinylidene difluoride (PVDF) membranes (Immobilon, Millipore). Immunoblotting was performed using monoclonal antibodies 4G10 (Upstate Biotechnology) against phosphotyrosine, HA.11 (Covance) against the HA-tag, B-5-1-2 (Abcam) against tubulin, and 8E9 against ABL. Immunoblots were visualized with SuperSignal West Pico (Pierce).

## Supporting Information

Figure S1 **BCR 14-3-3 binding and tyrosine 177 are not required to inhibit BCR-ABL nuclear import.** The indicated constructs of BCR-ABL proteins in the p185-BCR-ABL backbone were transiently expressed in murine embryo fibroblasts isolated from *Abl-null* mice, and their subcellular distribution was assessed by indirect immunofluorescence using the anti-ABL 8E9 antibody, without or with treatment with LMB (10 nM, 6 hr.). Detection of nuclear signals indicates re-activation of the NLS function by the specified mutations. The substitution of BCR Tyr177 with phenylalanine did not re-activate the NLS function, nor did the deletion of the BCR 14-3-3-binding site. The NLS function is re-activated in the kinase-defective p185-BCR-ABL. OD: oligomerization domain (BCR aa-1 to aa-63); GEF: guanine nucleotide exchange factor; PH: pleckstrin homology domain; C2: C2 domain binds calcium and phospholipids; Δ14-3-3 refers to the deletion of BCR aa-91 to aa-97, which binds the 14-3-3 adaptor protein; ND: not determined.
(TIF)

Figure S2 **BCR-ABL does not affect the nuclear import of ABL.** COS cells were transfected with HA-tagged BCR-ABL and GFP-tagged ABL expression constructs and treated without or with LMB (10 nM, 6 hr.). The anti-HA staining (red) shows the subcellular distribution of BCR-ABL, and the GFP (green) fluorescence shows the subcellular localization of ABL. Nuclei are counterstained with Hoechst dye (blue).
(TIF)

Figure S3 **Mutation of tyrosines 115, 185, 226, 264, 393 and 469 does not inhibit the NLS function of kinase-defective BCR_63_-ABL.** COS cells were transfected with a kinase-defective BCR_63_-ABL-6Y/F, in which six tyrosines in the kinase domain are mutated to phenylalanines as indicated in the schematic diagram (the amino acid numbering refers to that of ABL-1a). The phenylalanine substitutions of these six tyrosines did not inhibit the NLS function as indicated by the nuclear accumulation of BCR63-ABL-6Y/F after treatment with LMB (see nuclei marked by arrows). Nuclei were counterstained with Hoechst dye (blue).
(TIF)

Figure S4 **Imatinib binding re-activates the NLS function in kinase-defective BCR63-ABL with phenylalanine substitution at tyrosine 232, 253, 257.** The indicated constructs (KD: kinase-defective) were transfected into COS cells and the cells treated with LMB alone or LMB plus imatinib as indicated. Subcellular localization of the transiently transfected proteins was determined by indirect immunofluorescence staining with anti-ABL (8E9) antibody (red). DNA is counterstained with Hoechst dye (blue). Nuclear accumulation of the indicated kinase-defective BCR_63_-ABL-Y/F mutant protein was marked by white arrows.
(TIF)

Figure S5 **BCR63-ABL-Δ1121 does not co-localize with actin fibers.** The BCR63-ABL-Δ1121 protein was transiently expressed in COS cells. Immunofluorescence images of anti-ABL (8E9) staining (red) and F-actin stained with Alexa-488-conjugated phalloidin (green) are shown individually as well as merged (right most panel) with DNA staining by Hoechst dye (blue).
(TIF)

## References

[1] Lindgren V, Rowley JD (1977). Comparable complex rearrangements involving 8;21 and 9;22 translocations in leukaemia.. Nature.

[2] Shtivelman E, Lifshitz B, Gale RP, Canaani E (1985). Fused transcript of abl and bcr genes in chronic myelogenous leukaemia.. Nature.

[3] Pluk H, Dorey K, Superti-Furga G (2002). Autoinhibition of c-Abl.. Cell.

[4] Nagar B, Hantschel O, Young MA, Scheffzek K, Veach D (2003). Structural basis for the autoinhibition of c-Abl tyrosine kinase.. Cell.

[5] Van Etten RA (2003). c-Abl regulation: a tail of two lipids.. Curr Biol.

[6] McWhirter JR, Galasso DL, Wang JY (1993). A coiled-coil oligomerization domain of Bcr is essential for the transforming function of Bcr-Abl oncoproteins.. Mol Cell Biol.

[7] Zhao X, Ghaffari S, Lodish H, Malashkevich VN, Kim PS (2002). Structure of the Bcr-Abl oncoprotein oligomerization domain.. Nat Struct Biol.

[8] Taylor CM, Keating AE (2005). Orientation and oligomerization specificity of the Bcr coiled-coil oligomerization domain.. Biochemistry.

[9] Zhang X, Subrahmanyam R, Wong R, Gross AW, Ren R (2001). The NH(2)-terminal coiled-coil domain and tyrosine 177 play important roles in induction of a myeloproliferative disease in mice by Bcr-Abl.. Mol Cell Biol.

[10] He Y, Wertheim JA, Xu L, Miller JP, Karnell FG (2002). The coiled-coil domain and Tyr177 of bcr are required to induce a murine chronic myelogenous leukemia-like disease by bcr/abl.. Blood.

[11] Druker BJ, Sawyers CL, Kantarjian H, Resta DJ, Reese SF (2001). Activity of a specific inhibitor of the BCR-ABL tyrosine kinase in the blast crisis of chronic myeloid leukemia and acute lymphoblastic leukemia with the Philadelphia chromosome.. N Engl J Med.

[12] Druker BJ, Talpaz M, Resta DJ, Peng B, Buchdunger E (2001). Efficacy and safety of a specific inhibitor of the BCR-ABL tyrosine kinase in chronic myeloid leukemia.. N Engl J Med.

[13] Druker BJ, Tamura S, Buchdunger E, Ohno S, Segal GM (1996). Effects of a selective inhibitor of the Abl tyrosine kinase on the growth of Bcr-Abl positive cells.. Nat Med.

[14] Deininger MW, Goldman JM, Lydon N, Melo JV (1997). The tyrosine kinase inhibitor CGP57148B selectively inhibits the growth of BCR-ABL-positive cells.. Blood.

[15] le Coutre P, Tassi E, Varella-Garcia M, Barni R, Mologni L (2000). Induction of resistance to the Abelson inhibitor STI571 in human leukemic cells through gene amplification.. Blood.

[16] Weisberg E, Griffin JD (2000). Mechanism of resistance to the ABL tyrosine kinase inhibitor STI571 in BCR/ABL-transformed hematopoietic cell lines.. Blood.

[17] Mahon FX, Deininger MW, Schultheis B, Chabrol J, Reiffers J (2000). Selection and characterization of BCR-ABL positive cell lines with differential sensitivity to the tyrosine kinase inhibitor STI571: diverse mechanisms of resistance.. Blood.

[18] Gorre ME, Mohammed M, Ellwood K, Hsu N, Paquette R (2001). Clinical resistance to STI-571 cancer therapy caused by BCR-ABL gene mutation or amplification.. Science.

[19] Van Etten RA, Jackson P, Baltimore D (1989). The mouse type IV c-abl gene product is a nuclear protein, and activation of transforming ability is associated with cytoplasmic localization.. Cell.

[20] Wen ST, Jackson PK, Van Etten RA (1996). The cytostatic function of c-Abl is controlled by multiple nuclear localization signals and requires the p53 and Rb tumor suppressor gene products.. Embo J.

[21] Taagepera S, McDonald D, Loeb JE, Whitaker LL, McElroy AK (1998). Nuclear-cytoplasmic shuttling of C-ABL tyrosine kinase.. Proc Natl Acad Sci U S A.

[22] Vigneri P, Wang JY (2001). Induction of apoptosis in chronic myelogenous leukemia cells through nuclear entrapment of BCR-ABL tyrosine kinase.. Nat Med.

[23] Vella V, Zhu J, Frasca F, Li CY, Vigneri P (2003). Exclusion of c-Abl from the nucleus restrains the p73 tumor suppression function.. J Biol Chem.

[24] Henderson BR, Eleftheriou A (2000). A comparison of the activity, sequence specificity, and CRM1-dependence of different nuclear export signals.. Exp Cell Res.

[25] Fukuda M, Asano S, Nakamura T, Adachi M, Yoshida M (1997). CRM1 is responsible for intracellular transport mediated by the nuclear export signal.. Nature.

[26] Kudo N, Matsumori N, Taoka H, Fujiwara D, Schreiner EP (1999). Leptomycin B inactivates CRM1/exportin 1 by covalent modification at a cysteine residue in the central conserved region.. Proc Natl Acad Sci U S A.

[27] Wetzler M, Talpaz M, Van Etten RA, Hirsh-Ginsberg C, Beran M (1993). Subcellular localization of Bcr, Abl, and Bcr-Abl proteins in normal and leukemic cells and correlation of expression with myeloid differentiation.. J Clin Invest.

[28] McWhirter JR, Wang JY (1991). Activation of tyrosinase kinase and microfilament-binding functions of c-abl by bcr sequences in bcr/abl fusion proteins.. Mol Cell Biol.

[29] Daley GQ, Van Etten RA, Jackson PK, Bernards A, Baltimore D (1992). Nonmyristoylated Abl proteins transform a factor-dependent hematopoietic cell line.. Mol Cell Biol.

[30] Levinson NM, Kuchment O, Shen K, Young MA, Koldobskiy M (2006). A Src-like inactive conformation in the abl tyrosine kinase domain.. PLoS Biol.

[31] Nagar B, Bornmann WG, Pellicena P, Schindler T, Veach DR (2002). Crystal structures of the kinase domain of c-Abl in complex with the small molecule inhibitors PD173955 and imatinib (STI-571).. Cancer Res.

[32] Welch PJ, Wang JY (1995). Abrogation of retinoblastoma protein function by c-Abl through tyrosine kinase-dependent and -independent mechanisms.. Mol Cell Biol.

[33] Salomon AR, Ficarro SB, Brill LM, Brinker A, Phung QT (2003). Profiling of tyrosine phosphorylation pathways in human cells using mass spectrometry.. Proc Natl Acad Sci U S A.

[34] Steen H, Fernandez M, Ghaffari S, Pandey A, Mann M (2003). Phosphotyrosine mapping in Bcr/Abl oncoprotein using phosphotyrosine-specific immonium ion scanning.. Mol Cell Proteomics.

[35] Azam M, Latek RR, Daley GQ (2003). Mechanisms of autoinhibition and STI-571/imatinib resistance revealed by mutagenesis of BCR-ABL.. Cell.

[36] McWhirter JR, Wang JY (1993). An actin-binding function contributes to transformation by the Bcr-Abl oncoprotein of Philadelphia chromosome-positive human leukemias.. Embo J.

[37] Skourides PA, Perera SA, Ren R (1999). Polarized distribution of Bcr-Abl in migrating myeloid cells and co-localization of Bcr-Abl and its target proteins.. Oncogene.

[38] Hantschel O, Wiesner S, Guttler T, Mackereth CD, Rix LLR (2005). Structural basis for the cytoskeletal association of Bcr-Abl/c-Abl.. Molecular Cell.

[39] Wiesner S, Hantschel O, Mackereth CD, Superti-Furga G, Sattler M (2005). NMR assignment reveals an alpha-helical fold for the F-actin binding domain of human Bcr-Abl/c-Abl.. Journal of Biomolecular Nmr.

[40] Woodring PJ, Hunter T, Wang JY (2001). Inhibition of c-Abl tyrosine kinase activity by filamentous actin.. J Biol Chem.

[41] Brasher BB, Van Etten RA (2000). c-Abl has high intrinsic tyrosine kinase activity that is stimulated by mutation of the Src homology 3 domain and by autophosphorylation at two distinct regulatory tyrosines.. J Biol Chem.

[42] Tanis KQ, Veach D, Duewel HS, Bornmann WG, Koleske AJ (2003). Two distinct phosphorylation pathways have additive effects on Abl family kinase activation.. Mol Cell Biol.

[43] Smith KM, Yacobi R, Van Etten RA (2003). Autoinhibition of Bcr-Abl through its SH3 domain.. Mol Cell.

