# Supplementary figures and images for: Interplay between Kinase Domain Autophosphorylation and F-Actin Binding Domain in Regulating Imatinib Sensitivity and Nuclear Import of BCR-ABL
Source: PLoS One. 2011 Feb 11;6(2):e17020. doi: 10.1371/journal.pone.0017020 (PMC3037956; doi:10.1371/journal.pone.0017020)

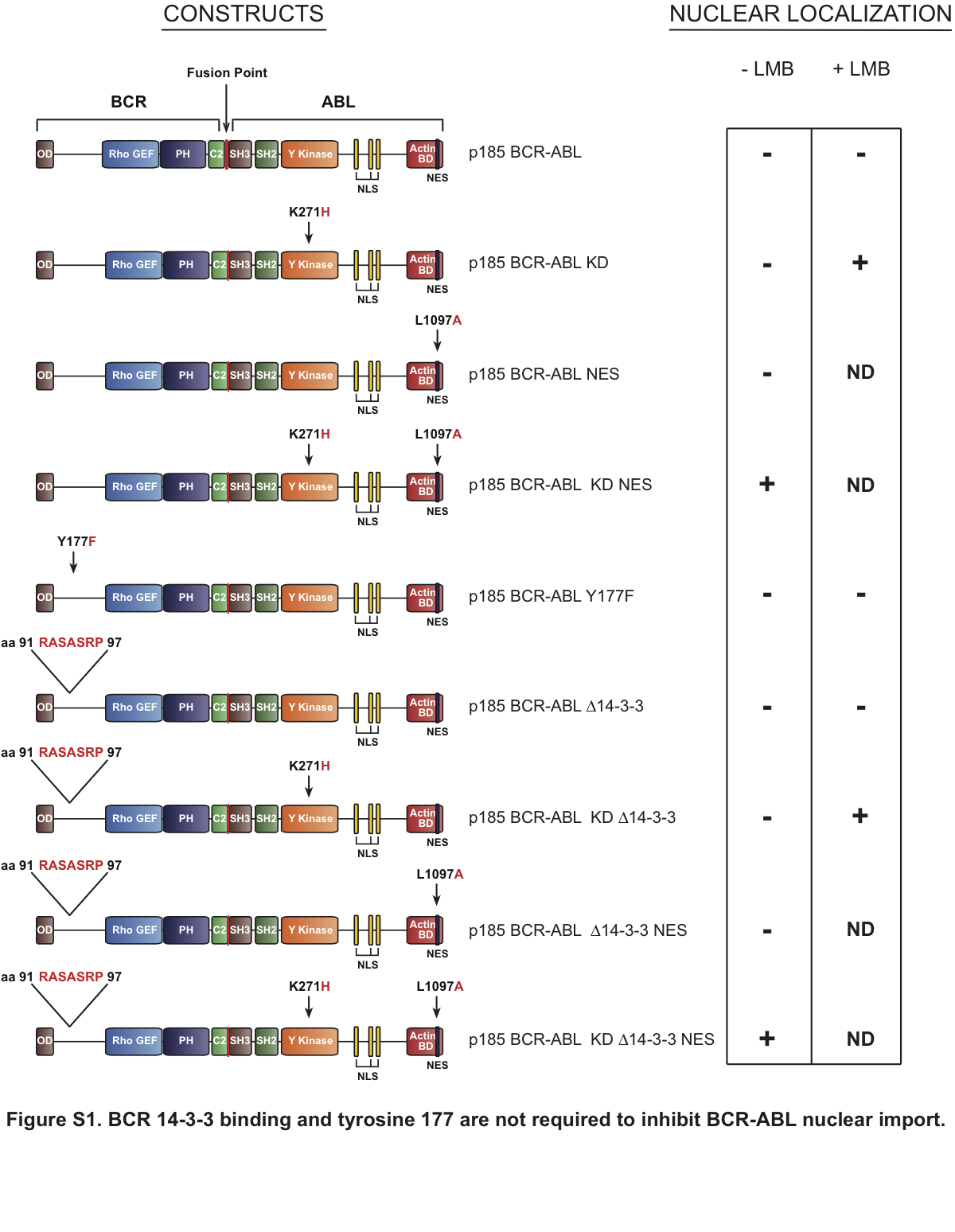

Supplement: Figure S1 — BCR 14-3-3 binding and tyrosine 177 are not required to inhibit BCR-ABL nuclear import. The indicated constructs of BCR-ABL proteins in the p185-BCR-ABL backbone were transiently expressed in murine embryo fibroblasts isolated from Abl-null mice, and their subcellular distribution was assessed by indirect immunofluorescence using the anti-ABL 8E9 antibody, without or with treatment with LMB (10 nM, 6 hr.). Detection of nuclear signals indicates re-activation of the NLS function by the specified mutations. The substitution of BCR Tyr177 with phenylalanine did not re-activate the NLS function, nor did the deletion of the BCR 14-3-3-binding site. The NLS function is re-activated in the kinase-defective p185-BCR-ABL. OD: oligomerization domain (BCR aa-1 to aa-63); GEF: guanine nucleotide exchange factor; PH: pleckstrin homology domain; C2: C2 domain binds calcium and phospholipids; Δ14-3-3 refers to the deletion of BCR aa-91 to aa-97, which binds the 14-3-3 adaptor protein; ND: not determined. (TIF) [file pone.0017020.s001.tif]

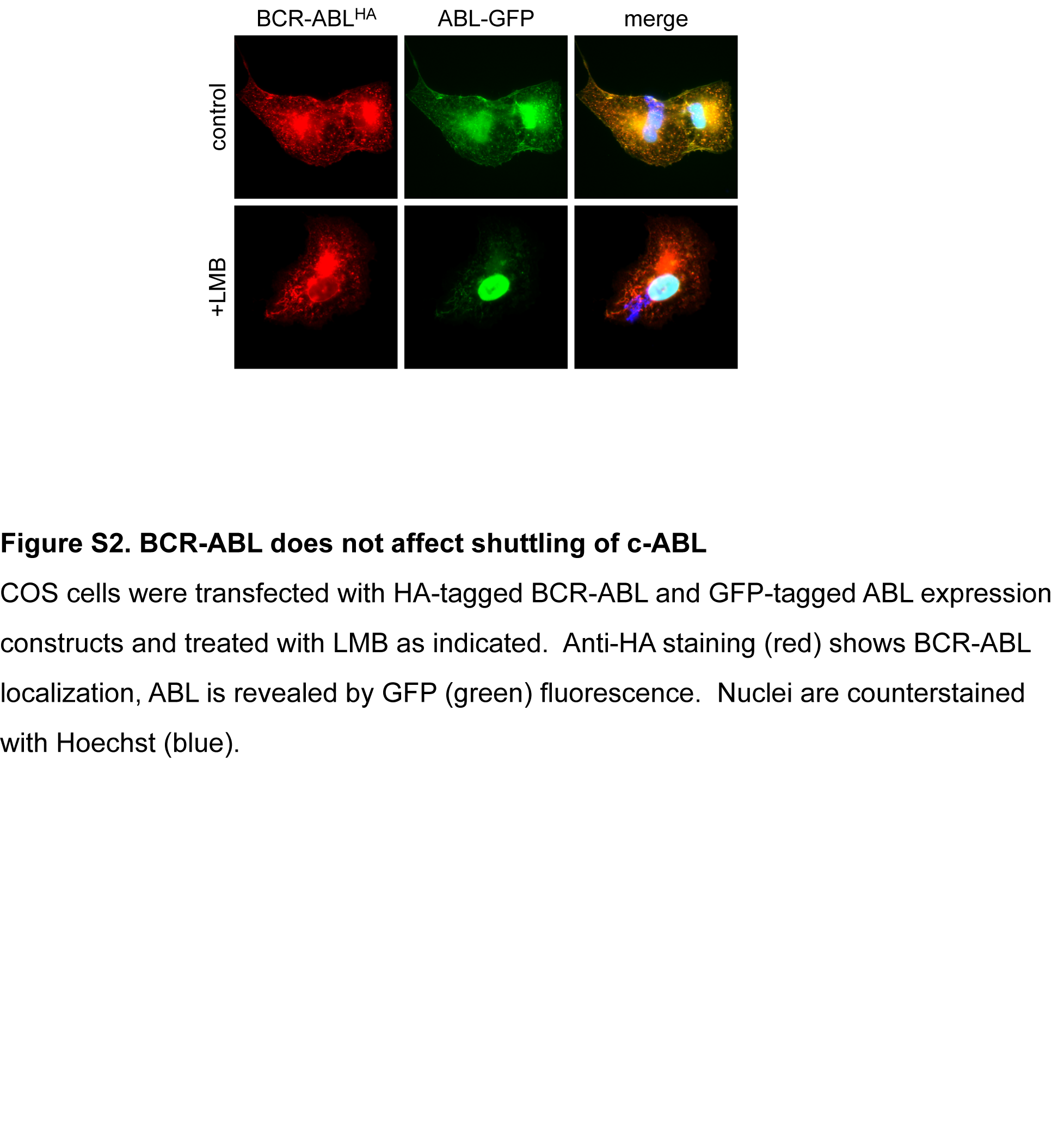

Supplement: Figure S2 — BCR-ABL does not affect the nuclear import of ABL. COS cells were transfected with HA-tagged BCR-ABL and GFP-tagged ABL expression constructs and treated without or with LMB (10 nM, 6 hr.). The anti-HA staining (red) shows the subcellular distribution of BCR-ABL, and the GFP (green) fluorescence shows the subcellular localization of ABL. Nuclei are counterstained with Hoechst dye (blue). (TIF) [file pone.0017020.s002.tif]

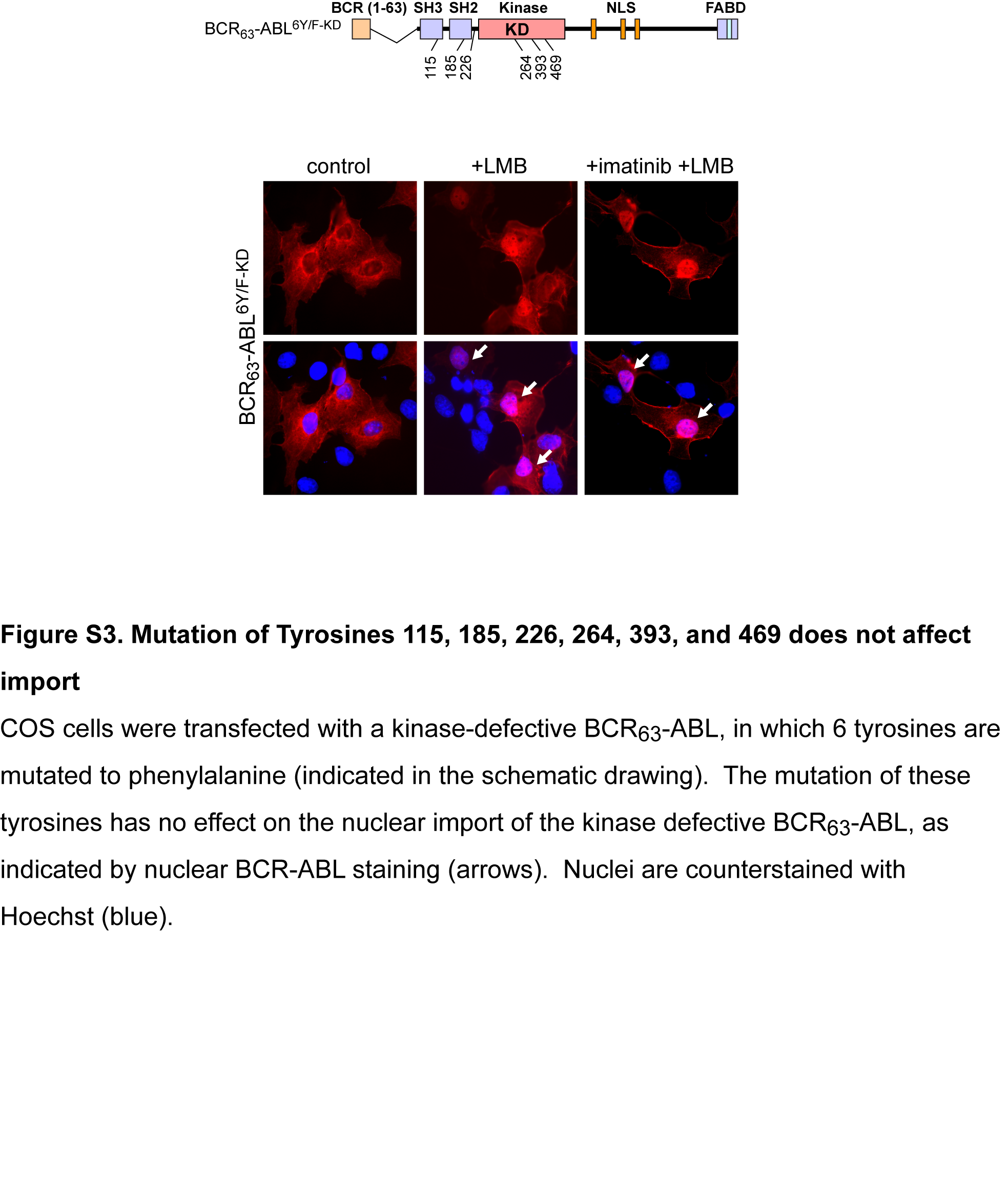

Supplement: Figure S3 — Mutation of tyrosines 115, 185, 226, 264, 393 and 469 does not inhibit the NLS function of kinase-defective BCR63-ABL. COS cells were transfected with a kinase-defective BCR63-ABL-6Y/F, in which six tyrosines in the kinase domain are mutated to phenylalanines as indicated in the schematic diagram (the amino acid numbering refers to that of ABL-1a). The phenylalanine substitutions of these six tyrosines did not inhibit the NLS function as indicated by the nuclear accumulation of BCR63-ABL-6Y/F after treatment with LMB (see nuclei marked by arrows). Nuclei were counterstained with Hoechst dye (blue). (TIF) [file pone.0017020.s003.tif]

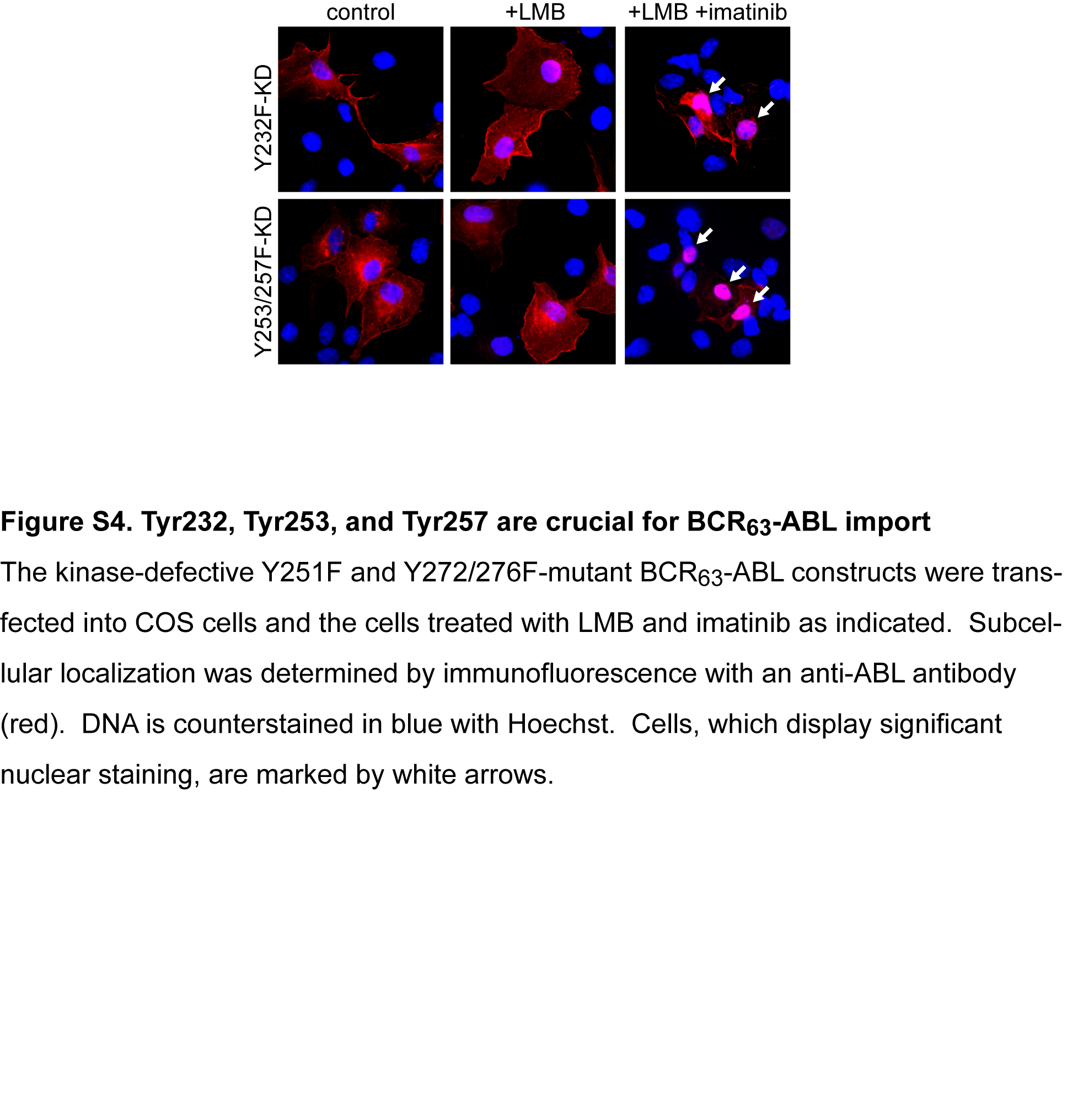

Supplement: Figure S4 — Imatinib binding re-activates the NLS function in kinase-defective BCR63-ABL with phenylalanine substitution at tyrosine 232, 253, 257. The indicated constructs (KD: kinase-defective) were transfected into COS cells and the cells treated with LMB alone or LMB plus imatinib as indicated. Subcellular localization of the transiently transfected proteins was determined by indirect immunofluorescence staining with anti-ABL (8E9) antibody (red). DNA is counterstained with Hoechst dye (blue). Nuclear accumulation of the indicated kinase-defective BCR63-ABL-Y/F mutant protein was marked by white arrows. (TIF) [file pone.0017020.s004.tif]

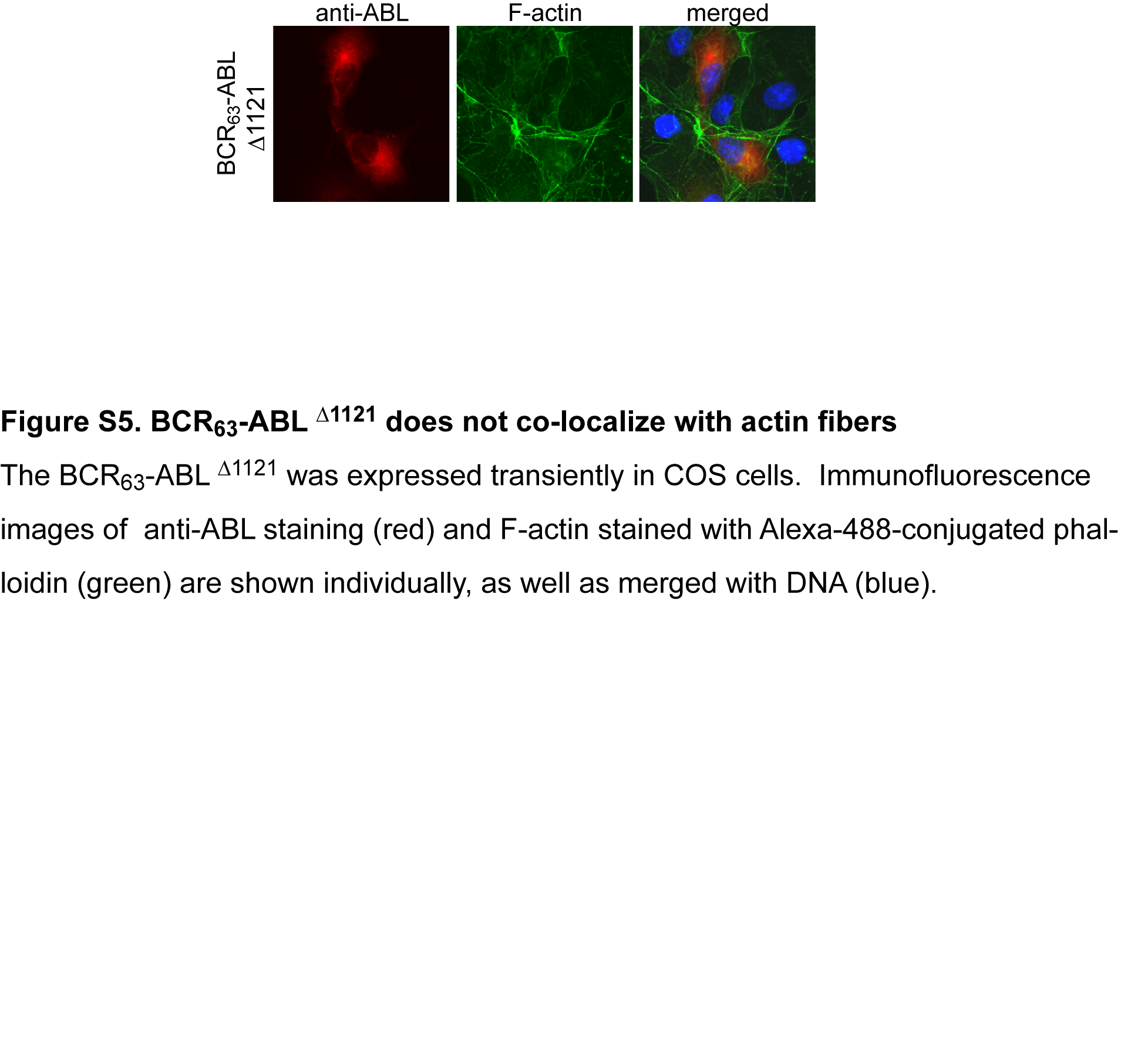

Supplement: Figure S5 — BCR63-ABL-Δ1121 does not co-localize with actin fibers. The BCR63-ABL-Δ1121 protein was transiently expressed in COS cells. Immunofluorescence images of anti-ABL (8E9) staining (red) and F-actin stained with Alexa-488-conjugated phalloidin (green) are shown individually as well as merged (right most panel) with DNA staining by Hoechst dye (blue). (TIF) [file pone.0017020.s005.tif]
